# Ethyl Protocatechuate Encapsulation in Solid Lipid Nanoparticles: Assessment of Pharmacotechnical Parameters and Preliminary In Vitro Evaluation for Colorectal Cancer Treatment

**DOI:** 10.3390/pharmaceutics15020394

**Published:** 2023-01-24

**Authors:** Stefano Russo, Cristina Torrisi, Nunzio Cardullo, Vera Muccilli, Alfonsina La Mantia, Francesco Castelli, Rosaria Acquaviva, Maria Grazia Sarpietro

**Affiliations:** 1 Department of Drug and Health Sciences, University of Catania, Viale Andrea Doria 6, 95125 Catania, Italy; 2 Department of Chemical Sciences, University of Catania, Viale Andrea Doria 6, 95125 Catania, Italy

**Keywords:** ethyl protocatechuate, SLN, colorectal cancer, CaCo-2 cells, DSC, encapsulation

## Abstract

Colorectal cancer is one of the most diffused tumoral diseases. Since most medicaments employed for its treatment are debilitating, the use of naturally derived products, which can be effective against the mutated cells and, in addition, can reduce most inflammatory-related effects, could be extremely beneficial for the continued treatment of this disease. In this research, ethyl protocatechuate (PCAEE), a protocatechuic acid prodrug, was encapsulated in solid lipid nanoparticles (SLN) (prepared without and with Tween 80), which were characterized in terms of size, polydispersity index (PDI), zeta potential and thermotropic behavior. Encapsulation efficiency, release profile and interaction with a model of biomembrane were also assessed. The nanoparticles were tested in vitro on both healthy cells and on a model of tumoral cells. SLN prepared with Tween 80 was promising in terms of physicochemical properties (z-average of 190 nm, PDI 0.150 and zeta potential around −20 mV) and encapsulation efficiency (56%); they showed a desirable release profile, demonstrated an ability to penetrate and release the encapsulated PCAEE into a biomembrane model and were nontoxic on healthy cells. In addition, they caused a greater dose-dependent decrease in the viability of CaCo-2 cells than PCAEE alone. In conclusion, the formulation could be proposed for further studies to assess its suitability for the treatment of colorectal cancer.

## 1. Introduction

Colorectal cancer (CRC) is one of the most diffused tumoral diseases, heavily connected to environmental and lifestyle-related causes [1], with worrying higher recurrence and death rates the more developed the cancer is and the more unfavorable the genetic analysis results are [2,3]. Screening and prevention have been demonstrated to be very effective in reducing the odds of the development of severe illness [1,4], while for the currently available treatments there is a lack of late-term efficacy on both physiological and psychological symptoms, since the illness itself and most medicaments too are strongly debilitating [5]. The occurrence of naturally derived products, which can be effective in killing the mutated cells and in reducing most inflammatory-related effects at the same time, could be extremely beneficial for the continued treatment of CRC.

**Scheme 1:**
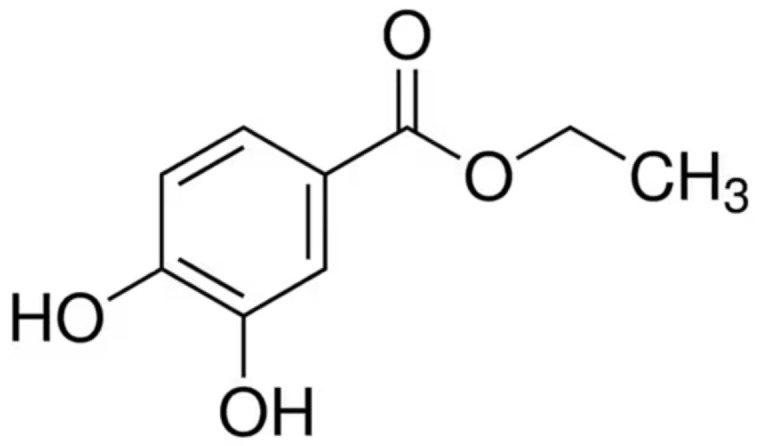
Chemical structure of ethyl protocatechuate.

Protocatechuic acid (PCA) is a catechol-like molecule with mixed antioxidant, anti-inflammatory and antiproliferative effects [6,7,8,9,10,11,12,13,14,15,16,17,18,19,20,21], probably due to its fairly simple structure and the consequent multi-target and off-target interactions [22]. Similar to other natural compounds, these multi-modal, sometimes “opposite” effects could be related to a state-dependent activity. The actual delivered dosage has been demonstrated to be a crucial factor in the therapeutic profile of PCA [23], since its metabolization is extensive even if dual-directional. Some of its metabolites are active while others are not, but in every case the excretion of both is fast due to rapid glucuronidation [6]. An effective way (largely used for other drugs) to extend the effects of PCA and to ameliorate its pharmacokinetics is to employ a prodrug [24] like ethyl protocatechuate (PCAEE) (Scheme 1), which has the added benefit of being a prolyl 4-hydroxylase inhibitor itself and thus being additionally used as a myocardium protector [25]. This is very useful for most patients surgically operated on for CRC who often suffer from myocardial postoperational complications [26]. To further improve PCAEE distribution and protection from early metabolization/excretion, inclusion in solid lipid nanoparticles (SLN) could be a promising strategy, since it has already been positively evaluated for skin delivery of PCA/PCAEE for advanced photoprotection strategy [27], making this type of carrier ideal for the job.

The aim of this study is to evaluate the encapsulation of PCAEE in SLN, to make thorough studies for the optimization of fundamental pharmacotechnical parameters of the obtained formulations through various techniques, including advanced DSC analyses and encapsulation/release assessment, and finally to preliminarily test the formulations in vitro on both healthy cells and an appropriate model (for CRC) of tumoral cells.

## 2. Materials and Methods

### 2.1. Materials

Protocatechuic acid ethyl ester was purchased from Sigma Aldrich Chimica s.r.l. (Milan, Italy). Precirol^®^ ATO 5 (glyceryl distearate) and Gelucire 50/13 were gifted by Gattefossé (Saint-Pries, France). Tween^®^ 80 (polysorbate 80) was retrieved from Sigma Aldrich Co. (St. Louis, MO, USA). The chemical 1,2-dimyristoyl-sn-glycero-3-phosphocholine (DMPC) was purchased from Genzyme (Liestal, Switzerland).

The chemical 3-(4,5-dimethyl-2-thiazolyl)-2,5-diphenyl-2H-tetrazolium bromide (MTT), Dulbecco’s modified Eagle’s medium, fetal bovine serum, glucose, and penicillin–streptomycin, were obtained from Sigma Aldrich (Milan, Italy).

Human foreskin fibroblast (HFF-1) and human colorectal adenocarcinoma (CaCo-2) cells were bought from ATCC^®^ SCRC-1041 TM (ATCC, Manassas, VA, USA). Purified water from Millipore-Q^®^ Gradient A10TM ultra-pure water system (Millipore, Guyancourt, France) was produced as needed and employed for the duration of the studies.

### 2.2. SLN Preparation

SLN were prepared with and without Tween 80; unloaded SLN and SLN loaded with PCAEE were also prepared. The formulations’ names and their composition are reported in Table 1. The preparation of SLN is based on the phase inversion temperature–ultrasonication combined method, described elsewhere [28]. Briefly, 500 mg of PrecirolATO 888 (T_m_ = 55 °C) and 200 mg of Gelucire 50/13 were transferred in a beaker and successively placed in a water bath onto a hotplate magnetic stirrer. The temperature was set 20 °C higher than the melting point of the main lipid component. Once the mix began to melt, a gentle stirring (100 rpm) was performed, and, for PCAEE-loaded SLN, 1 mL of dichloromethane was poured in, to ensure the compound solubilization in the lipids, and then allowed to evaporate. Twenty milliliters of MilliQ Water were heated at the same temperature used in the lipid phase and then transferred drop by drop in the lipid phase under mild agitation (250 rpm). After that, the obtained emulsion was allowed to rest for 3 min. Soon after, the emulsion was processed with a UP400S probe ultrasonicator (Ultra-Schallprozessor, Dr. Hielscher GmbH, Teltow, Germany) for 6 min at 50% amplitude (200 W). Finally, the formulation was cooled at room temperature and brought to volume (20 mL) with MilliQ Water. For SLN prepared with Tween 80, 190 µL of Tween 80 (1% *w*/*w*) were added after the samples were brought to volume, followed by 30 s of vortexing: the concentration employed assured the surfactant effect along with the absence of surfactant-related toxicity [29,30]. For the PCAEE-loaded preparations, 10 mg of PCAEE were added to the lipid mixture from the start.

### 2.3. SLN Characterization

Mean particle size (z-average) and polydispersity index (PDI) of formulations were evaluated through dynamic light scattering (DLS) by usage of a Zetasizer Nano-ZS90 (Malvern Instrument Ltd., Worcs, UK), supplied with a solid-state laser, with a 4.5 mW nominal output and a 5 mW maximum power at 670 nm. Analyses were developed with the following settings: 90° scattering angle at 25 ± 0.2 °C. Zeta potential (ZP) was extrapolated by means of the electrophoretic light scattering (ELS) technique, which evaluates the electrophoretic mobility of particles, relatable to stability, in a dispersion. Each sample was prepared by dilution of 100 µL of SLN suspension into 900 µL of MilliQ water, and analyses were conducted in triplicate. The morphologies were investigated through a field emission scanning electron microscopy (FE-SEM) ZEISS SUPRA 55 VP (White Plains, NY, USA). All the FE-SEM images were recorded operating at 10 kV and with a working distance of 5.5 mm, using the in-lens secondary electron detector.

### 2.4. Encapsulation Efficiency

SLN-PCAEE and SLN-T-PCAEE formulations (0.4 mL) were loaded onto a column (1.0 × 10 cm) packed with Sephadex LH20 and eluted with 15 mL of water, 10 mL of EtOH, and finally 10 mL of acetone [28]. The fractions were analyzed by HPLC-UV equipped with a Luna C-18 column (Phenomenex; 250 mm × 4.60 mm, 5 µM), employing the following elution program made up of H_2_O+ 1%CH_2_O (solvent A) and CH_3_CN+ 1%CH_2_O: t0 = 0% B, t_10_ = 50% B, t_15_ = 100% B, t_25_ = 0% B. The flux was set at 1.0 mL/min and the DAD at 254 nm. A PCAEE pure sample (purity > 98%) was injected in these chromatographic conditions (t_R_ = 12.365 min) and employed for the identification of the molecule in the fractions. PCAEE quantification was achieved with an external standard calibration curve in the range of 0.05 mg/mL to 1.0 mg/mL (r^2^ = 0.9990). In the aqueous fraction of Sephadex LH-20, separation eluted the PCAEE loaded SLNs, and, conversely, in the ethanol fraction, it eluted the free PCAEE. The entrapment efficiency % (EE%) was determined with Equation (1)

EE% = [(mgPCAEE_tot_ − mgPCAEE_free_) ÷ mgPCAEE_tot_] × 100 (1)

The procedure was repeated three times, and data were expressed as mean (*n* = 3) ± SD.

### 2.5. PCAEE Release from SLN

In vitro release study of PCAEE was assessed by employment of 3.5 kDa cut-off dialysis tubes (Spectra/Pro, Spectrum Lab., Compton, CA, USA). One milliliter of each sample was poured into a dialysis tube, which was then inserted in a beaker containing 30 mL of 50 mM TRIS buffer (pH 7.4).

The solution was stirred at 200 rpm and 37 ± 0.5 °C. One milliliter of the release medium was withdrawn at certain time points and substituted with an equal volume of fresh release medium. Samples were lyophilized and then examined by HPLC–UV with the same chromatographic conditions used for the entrapment efficiency determination.

### 2.6. Multilamellar Vesicles Preparation

A DMPC solution in chloroform/methanol (1:1, *v*:*v*) was prepared. The solvents were evaporated under nitrogen flow, and the resulting film was lyophilized to eliminate solvent residues. A quantity of 50mMTris solution (pH = 7.4) was added to the films to have 244 µmol/mL of phospholipid. The sample was heated at 37 °C for 1 min and successively shaken for 1 min. This procedure was repeated three times. Finally, the sample was kept at 37 °C for 1 h [31].

### 2.7. DSC Analysis

A Mettler Toledo STARe system (Mettler Toledo, Greifensee, Switzerland), equipped with a DSC-1 calorimetric cell and Mettler TA-STARe software (version 16.00), was used. The sensitivity was automatically chosen as the maximum possible by the calorimetric system. The reference pan was filled with TRIS 50 mM solution. To calibrate the system in temperature and enthalpy changes, the standard procedure for the DSC-1 Mettler TA STARe instrument was followed.

#### 2.7.1. SLN and MLV Analysis

To evaluate the thermotropic behavior of the SLN, the formulation was submitted to DSC analysis under N2 flow (70 mL/min) as follows: a heating scan from 5 to 85 °C, at 2 °C/min, and a cooling scan from 85 to 5 °C, at 4 °C/min, at least three times to confirm the reproducibility of data [32]. MLV were analyzed by the same procedure.

#### 2.7.2. MLV-SLN Interaction Analysis

An amount of 30 µL of MLV and of 90 µL of SLN or SLN-PCAEE were placed in a 160 µL DSC aluminum pan, which was hermetically sealed and subjected to calorimetric analysis under N_2_ flow (70 mL/min) as follows: (1) a heating scan from 5 to 85 °C at the rate of 2 °C/min, (2) a cooling scan from 85 to 37 °C at the rate of 4 °C/min, (3) an isothermal period of one hour at 37 °C, and (4) a cooling scan from 37 to 5 °C (4 °C/min). This procedure was repeated eight times [31].

### 2.8. Cell Culture

CaCo-2 cells were cultured in Dulbecco’s modified Eagle’s medium with 10% fetal calf serum, 1 mmol/L sodium pyruvate, 2 mmol/L L- glutamine, streptomycin (50 mg/mL), and penicillin (50 U/mL). HFF-1 cells, used as control, were cultured in Dulbecco’s modified Eagle’s medium supplemented with 15% fetal bovine serum, 4.5 g/L glucose, 100 U/mL penicillin, and 100 μg/mL streptomycin.

### 2.9. MTT Bioassay

The MTT assay, used to assess cell viability, measures the conversion of tetrazolium salt to colored formazan in the presence of metabolic activity, as previously reported [33]. The amount of formazan is proportional to the number of living cells. A microplate spectrophotometer reader (Titertek Multiskan, Flow Laboratories, Helsinki, Finland) was used to measure the optical density of each well sample at λ = 570 nm.

CaCo-2 and HFF-1 cells, seeded in 96-well constant density microplates (8 × 10^3^ cells/well), after 24 h incubation in a humidified atmosphere of 5% CO_2_ at 37 °C, were treated with different concentrations of either SLN-T (1:25-1:50-1:100-1:200 dilution ratios), free PCAEE (12.5–25–50-100 μM) or SLN-T-PCAEE (at the same dilution ratios of SLN-T, which correspond to the free PCAEE concentrations) for 72 h. Four replicates were performed for each sample. For CaCo-2 cells, doxorubicin was used as a standard of cytotoxicity. The results are expressed as a percentage of cell viability compared with untreated cells (Ctr). The controls are untreated control cells (Ctr) for SLN, PCAEE and SLN-T-PCAEE, and PCAEE-treated cells for SLN-T-PCAEE.

### 2.10. Statistical Analysis

Statistical analysis was performed using GraphPad Prism 9.0.0 software (Boston, MA, USA); the algorithm employed was the ordinary two-way ANOVA along with multiple group comparisons corrected by Tukey test method.

## 3. Results and Discussion

### 3.1. SLN Characterization

**Figure 1:**
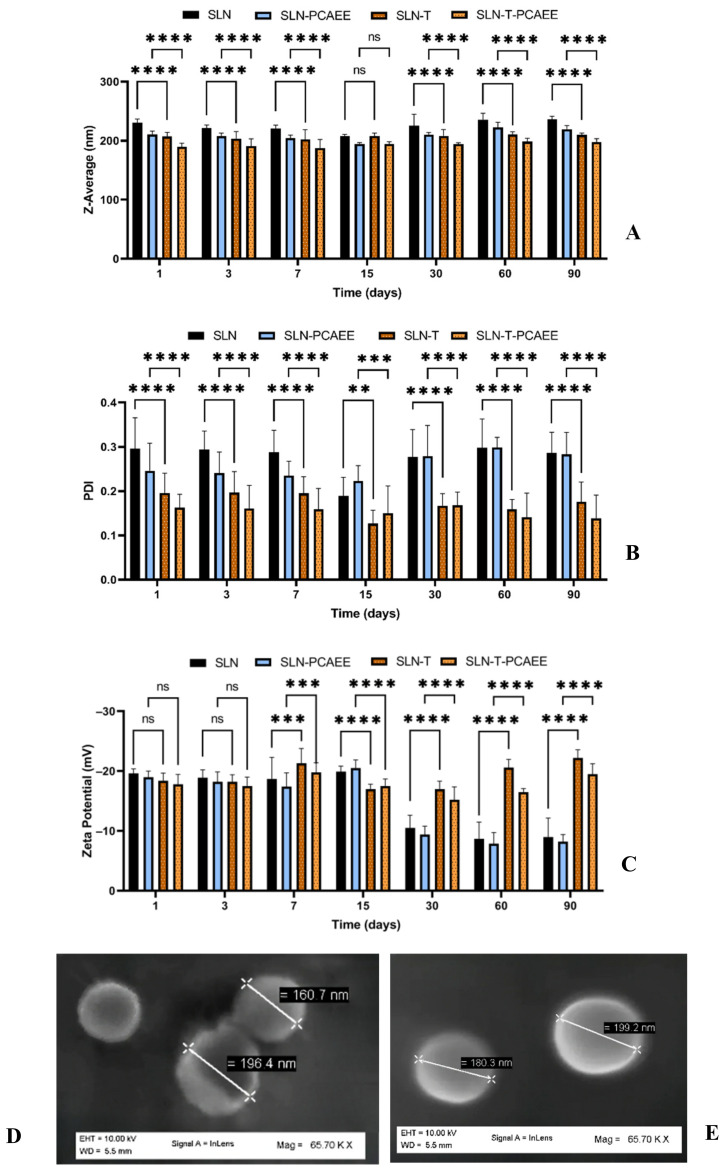
(**A**) Mean particle size (z-average), (**B**) polydispersity index (PDI), (**C**) zeta potential of SLN. The results are shown as mean ± standard deviation. Confidence intervals calculated by two-way ANOVA: ns = not significant; ** = *p* < 0.01; *** = *p* < 0.001; **** = *p* < 0.0001. (**D**) SEM images of SLN-T and (**E**) SEM images of SLN-T-PCAEE.

Characterization and evaluation of the physiochemical stability of the SLN was obtained by mean particle size, polydispersity index (PDI), zeta potential analyses over three months at 25 °C and SEM images (Figure 1A–E). SLN exhibited a mean particle size of 230 nm, which remained unchanged over the period; the inclusion of PCAEE caused a size decrease. SLN-T showed a mean particle size of about 200 nm, which remained stable over the entire period. PCAEE inclusion caused a slight mean particle size decrease. PDI values ranged over time from ~0.3 for SLN to ~0.11 for SLN-T-PCAEE.

Zeta potential of SLN and SLN-PCAEE harshly decreased after 15 days, while for SLN-T and SLN-T-PCAEE zeta potential underwent an initial decrease but after 30 days they gradually stabilized around −20 mV.

SEM images of the SLN-T and SLN-T-PCAEE showed uniform and spherical shape of the particles, with average dimensions consistent with DSL measurements.

The results obtained suggest that SLN-T are more suitable for PCAEE encapsulation and delivery. The obtained nanoparticles, due to the surfactant effect of Tween 80, displayed better results, compared with the preparations without surfactant, in terms of dimensions and polydispersity and higher absolute zeta potential values, which favor the separation between nanoparticles by prevention of their aggregation caused by electrical interactions [34].

### 3.2. Encapsulation Efficiency

The methodology, previously developed [28] on the basis of Sephadex-LH20 column chromatography followed by HPLC identification and quantification method, was adapted for the determination of the entrapment efficiency (EE%) of PCAEE in SLN and SLN containing Tween 80 (SLN-T). The EE% was obtained indirectly (Equation 1) by quantification of free PCAEE eluted with ethanol from gel permeation chromatography. The results indicated an EE% = 80% ± 4 in SLN-PCAEE and an EE% = 56% ± 2 in SLN-T-PCAEE. The higher encapsulation obtained for SLN without Tween cannot justify the employment of these carriers, since they were demonstrated to be toxic, even empty SLN, for both the cellular lines employed in this study (data not shown), likely for an occlusive effect on the cell membrane caused by SLN aggregation in absence of surfactant and for higher lipid-related oxidative damage [35,36]. Moreover, higher dosages of PCAEE included in SLN without Tween 80 could negatively affect the pharmacokinetic profile of the drug (too slow release, absence of initial burst effect), rendering SLN-T more suitable as transporters.

### 3.3. PCAEE Release from SLN-T

**Figure 2:**
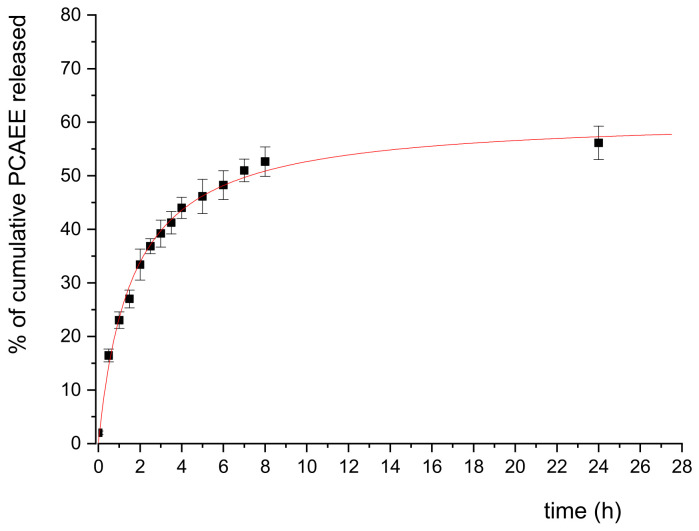
In vitro release profile of PCAEE from SLN in TRIS buffer. The data plotted are means (*n* = 3) ± SD and were fitted with a hyperbolic curve.

The release of PCAEE from SLN-T was simulated in vitro employing the dialysis method in TRIS buffer (50 mM, pH 7.4). According to a preliminary experiment, samples were collected every 30 min for the first 4 h, every 1h up to 8 h, and a final one after 24 h. The PCAEE released was quantified by HPLC-UV, and the results are reported in Figure 2 as percent of cumulative PCAEE released vs time. The release in TRIS buffer is faster in the first 5 h, reaching up to 46% of total PCAEE; this result is explained by the immediate crossing of free and adsorbed-to-SLNs PCAEE (according to the 56% EE) through the dialysis tube. The release slows down between 5 and 8 h (52% at 8 h) and proves to be constant at 24 h (56%), indicating a continuous liberation of the encapsulated PCAEE fraction. The release profile was successfully fitted to a hyperbolic curve, indicating a burst effect in the first 3 h, a steady release between hours 3 and 8 and a steady, long-term liberation of the drug after 8 h; the profile obtained is desirable for the proposed application of PCAEE.

### 3.4. DSC Analysis

#### 3.4.1. SLN and MLV Analysis

**Figure 3:**
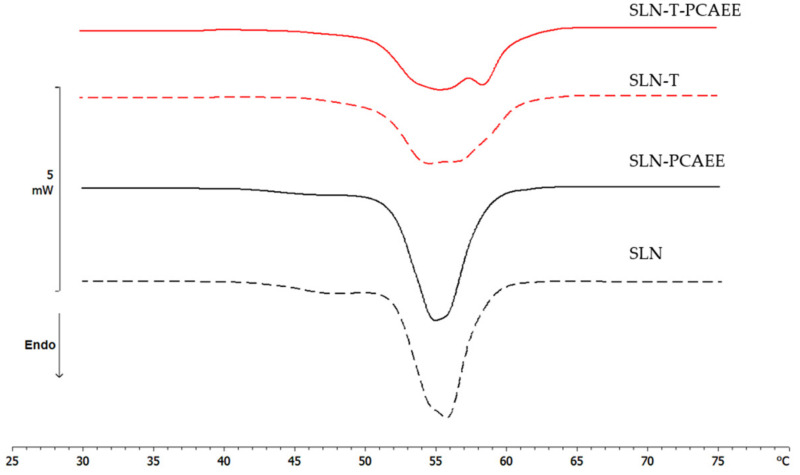
Calorimetric curves, in heating mode, of SLN, SLN-PCAEE, SLN-T and SLN-T-PCAEE.

Differential scanning calorimetry is a powerful technique to study the thermotropic behavior of SLN [28,37]. Calorimetric curves are shown in Figure 3. SLN curve is characterized by a first large shoulder at about 47 °C, a second shoulder at 54 °C and a main peak at 55.78 °C. The SLN-PCAEE curve shows a first shoulder (less intense than SLN), a peak at 54.99 °C and a second shoulder at higher temperature. SLN-T presents a multi-peak curve with the main one at 54.12 °C and a shoulder at higher temperature. In SLN-T-PCAEE, the three peaks are still present, but with a different morphology; in particular, the shoulder at ~59 °C becomes a well-defined peak. Both SLNs with PCAEE display lower enthalpies compared with empty SLNs. These data demonstrate that PCAEE largely modify their thermotropic behaviour by inclusion in the lipid matrix.

**Scheme 2:**
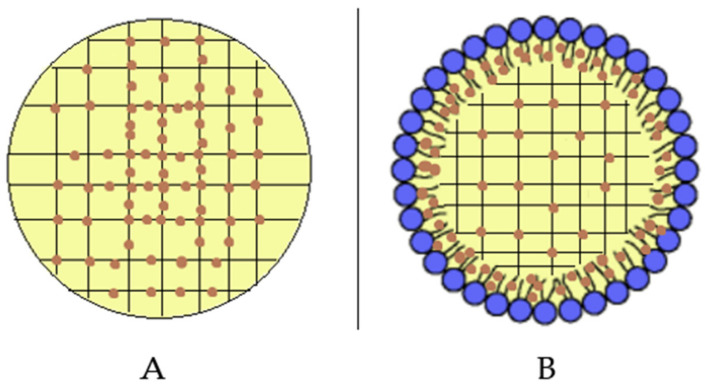
Localization of PCAEE (orange dots) in (**A**) SLN-PCAEE and (**B**) SLN-T-PCAEE. Blue dots with short black lines represent Tween 80.

A comparison between SLN and SLN-T should be made. The use of Tween 80 for SLN preparation led to an enlargement of the peak, a reduction of the enthalpy variation from −164 J/g to −123 J/g and an increase of the ΔT_1/2_ from 3.92 °C to 6.81 °C. This could be due to a shell-located deposition of Tween 80 on the SLN surface and partial insertion of the polymeric chains into the SLN. This phenomenon causes a decrease in the cooperation between Precirol molecules during the melting. In SLN-T-PCAEE the drug could then mainly be located among the portion of the Tween 80 chains inserted in the SLN shell, leaving the Precirol organization almost unaltered (Scheme 2).

**Figure 4:**
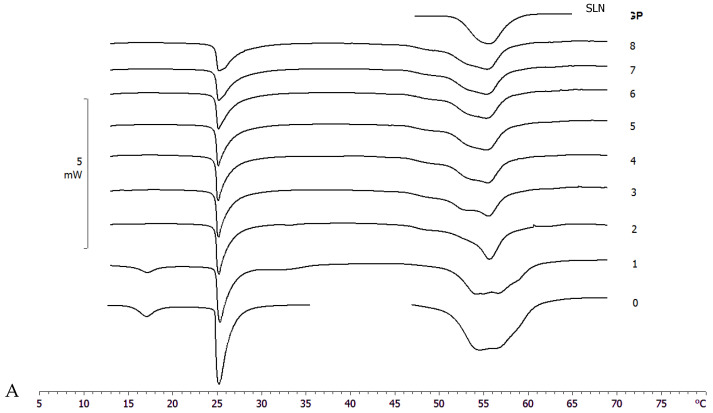

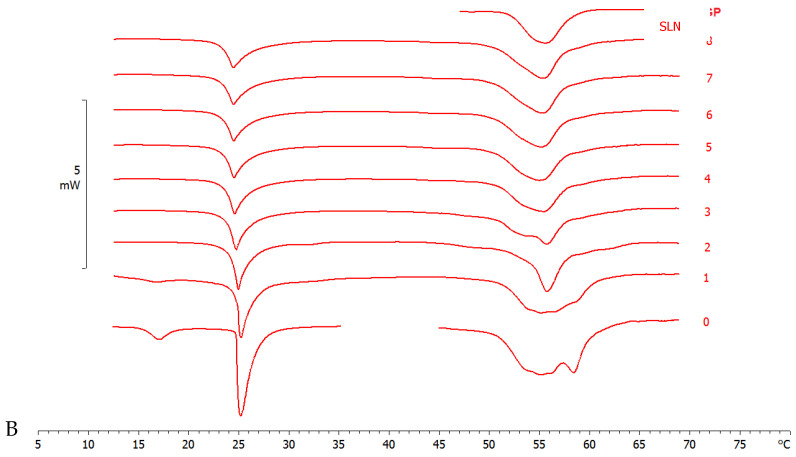
Calorimetric curves, in heating mode, of (**A**) MLV and SLN-T and (**B**) MLV and SLN-T-PCAEE. Curve 0 refers to MLV and nanoparticles before the contact. Curves 1–8 refer to calorimetric curves recorded at increasing contact time.

The DMPC MLV calorimetric curve shows a pre-transition peak at 17 °C, due to the transition from the ordered-gel phase to the ripple phase, and a main peak at 25.09 °C related to the transition from the ripple phase to the disordered-liquid crystalline phase (Figure 4) [38].

#### 3.4.2. MLV-SLN Interaction Analysis

The interaction of SLN-T and SLN-T80 with MLV (used as a biomembrane model) was studied by DSC analysis. MLV and nanoparticles were put in contact in the calorimetric pan and submitted to DSC analysis. The interaction between the two systems is demonstrated by the variation of their calorimetric parameters (transition temperature and/or enthalpy variation). In Figure 4A, the calorimetric curves of MLV and SLN-T are shown. The pretransition peak of MLV is lost while the main peak decreases with a gradual variation of the enthalpy change (from −25.55 J/g in the first scan to −15.68 J/g in the last scan). A deep variation occurs in the SLN-T signal, and the large multipeak signal gradually changes to a sharp peak preceeded by two shoulders. This behaviour clearly indicates that an interaction between MLV and SLN-T occurs. A similar trend is shown when MLV and SLN-T-PCAEE are put in contatct (Figure 4B). The pretransition peak of MLV disappears and the main peak decreases in intensity and enlarges, hinting of the suppressed cohesive interactions between adjacent DMPC molecules. The SLN-T-PCAEE signal becomes sharper and loses the multipeak feature. An interesting piece of evidence is that in the last scans the calorimetric signal of SLN-T, and even more so SLN-T-PCAEE, is similar to that of SLN. The hypothesis is that as the contact between MLV and SLN-T occurs, Tween 80 moves from the SLN-T to the MLV surface and then moves among the DMPC molecules, decreasing the enthalpy variation and facilitating the interaction. Similarly, Tween 80 and PCAEE, in accordance with the cooperation hypothesis presented earlier, could transfer from SLN-T-PCAEE to MLV surface and gradually into the bilayers among the DMPC molecules.

### 3.5. Effects of SLN with Tween 80 on Cell Viability

**Figure 5:**
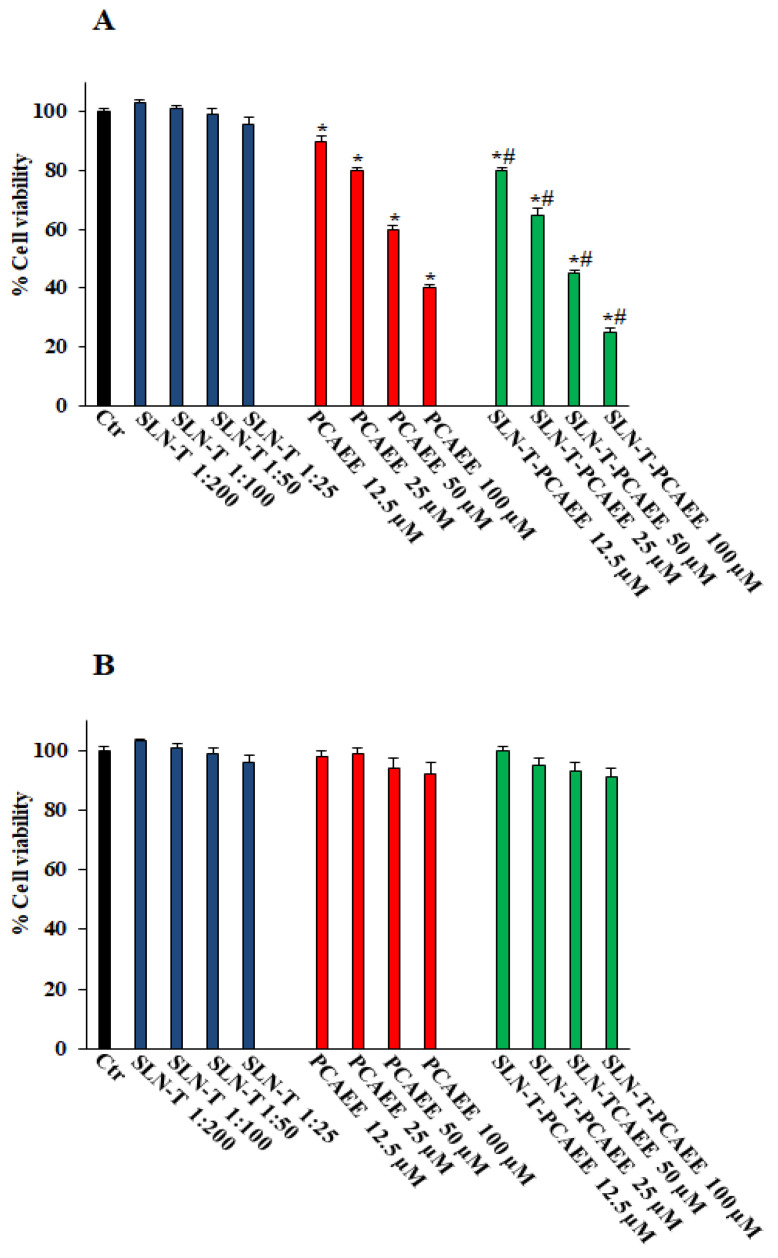
Cell viability in CaCo-2 (**A**) and HFF-1 (**B**) untreated (Ctr) and treated with empty SLN-T, PCAEE and SLN-T-PCAEE at different concentrations. Values are expressed as mean ± S.D. of four experiments in triplicate. * Significant vs. untreated control cells: *p* < 0.001; # Significant vs. PCAEE-treated cells: *p* < 0.001.

Figure 5A shows that pretreatment with SLN-T-PCAEE for 72 h induces, in a dose-dependent manner, a decrease in the viability of CaCo-2 cells compared with those treated with empty SLN-T, with PCAEE alone and the control group. In particular, the effect of SLN-T-PCAEE is higher than PCAEE alone, and at a concentration of 100 μM it reduces cell viability by ~80% compared with the ~57% induced by treatment with PCAEE alone (IC50 doxorubicin, 21 ± 0.3 μM). Treatment with PCAEE, SLN-T and SLN-T-PCAEE showed no toxicity on HFF-1 cells under the same experimental conditions (Figure 5B). All of the IC50 values are reported in Table 2. The MTT test was also performed at 24 and 48 h, but no significant change in cell viability was detected. These results confirmed that SLN-T-PCAEE formulation induces an optimal release of PCAEE and that the encapsulation of PCAEE with SLN-T enhanced its effect, because PCAEE alone has a worse permeation through the cell membrane compared with SLN-T-PCAEE. The data also suggest a selectivity of the drug against cancerous cells, leaving normally functioning cells intact at the same concentrations, which is not altered by the encapsulation in the nanoparticles.

## 4. Conclusions

The aim of this research was to encapsulate ethyl protocatechuate into solid lipid nanoparticles to be used for colorectal cancer treatment and to optimize the fundamental pharmacotechnical parameters of the obtained nanoparticles through various techniques, including advanced DSC analyses and encapsulation/release assessment, and to test the formulations in vitro on both healthy cells and an appropriate model of tumoral cells. SLN with Tween 80 and SLN without Tween 80 were prepared. Both SLNs showed promising results in terms of physicochemical properties and encapsulation efficiency, but SLN prepared without Tween was shown to be cytotoxic. Empty SLN prepared with Tween 80 proved to be safe, as it did not affect the cellular viability. In addition, SLN-T-PCAEE displayed a desirable release profile and the ability to penetrate and liberate the encapsulated PCAEE into a biomembrane model. SLN-T-PCAEE caused a greater dose-dependent decrease in the viability of CaCo-2 cells than did PCAEE alone, while not interfering with healthy cells, as represented by the HFF-1 model. The present work is a preliminary, mainly technological characterization study that could be considered the starting point for considering such nanoparticles as useful delivery systems for PCAEE. In conclusion, the formulation could be proposed as a candidate for further studies to assess its suitability for the treatment of colorectal cancer. Other biological experiments are programmed for the subsequent article regarding this formulation. A series of biological and biochemical assays, including nonmitochondrial cellular vitality tests, such as immunocytochemistry and flow cytometry, will surely be given strong consideration. In addition, inclusion of nanoparticles in either a hydrogel or an enema for rectal application will be evaluated.

## Figures and Tables

**Table 1:** SLN composition.

Sample	PrecirolATO 888 (mg)	Gelucire 50/13 (mg)	Tween 80 (µL)	PCAEE (mg)	H_2_O
SLN ^a^	500	200	----	----	up to 20 mL
SLN-PCAEE ^b^	500	200	----	10	up to 20 mL
SLN-T ^c^	500	200	190	----	up to 20 mL
SLN-T-PCAEE ^d^	500	200	190	10	up to 20 mL

^a^ unloaded SLN; ^b^ SLN loaded with PCAEE; ^c^ unloaded SLN prepared with Tween 80; ^d^ SLN prepared with Tween 80 and loaded with PCAEE.

**Table 2:** IC50 values. Values are the mean ± S.D. of four experiments in triplicate. N.D. = not determined.

Cell Line	Treatment	MTT Test IC_50_ (μM)
CaCo-2	SLN-T	N.D.
CaCo-2	PCAEE	82.2 ± 1.7
CaCo-2	SLN-T-PCAEE	45.7 ± 2.3
CaCo-2	Doxorubicin	21.0 ± 0.3
HFF-1	SLN-T	N.D.
HFF-1	PCAEE	N.D.
HFF-1	SLN-T-PCAEE	N.D.
HFF-1	Doxorubicin	N.D.

## Data Availability

Data were generated at Department of Drug and Health Science and Department of Chemical Sciences, University of Catania. Data supporting the results of this study are available from the corresponding author on request.
